# Financial problems for underprivileged people undergoing liver transplantation procedure in Pakistan

**DOI:** 10.1016/j.amsu.2022.104052

**Published:** 2022-06-23

**Authors:** Kaleem Ullah, Abdul Wahab Dogar, Husna Nisar, Sidhant Ochani, Afnan W.M. Jobran, Muhammad Wajeeh Nazar

**Affiliations:** a Department of Liver Transplantation, Pir Abdul Qadir Shah Jeelani Institute of Medical Sciences, Gambat, Pakistan; b Mehboob School of Physiotherapy, Peshawar, Pakistan; c Khairpur Medical College, Khairpur Mir's, Pakistan; d Faculty of Medicine, Al Quds University, Jerusalem, Palestine; e CMH Multan Institute of Medical Sciences, Pakistan

Respected Editor,

Liver transplantation is the primary surgical treatment for treating end-stage liver disease patients having various etiologies and hepatocellular carcinoma. Liver transplantation provides the best opportunity for the survival of these patients [1]. Liver transplantation is a major procedure and it not only requires the highest level of technical expertise but also needs various equipments and intensive care units [2]. Those who are offered liver transplantation have always various queries in their minds like the procedure cost, success rate, life expectancy, and post-procedure quality of life [3].

Pakistan is a developing country and its healthcare system is facing serious challenges like sacristy of resources, insufficient and non-trained human resources, and systemic mismanagement [4]. The living donor liver transplantation procedures in Pakistan cost approximately USD 45,000 [2]. Due to financial constraints and limited resources, the majority of patients from such low-income countries cannot afford the expensive treatment procedures [3]. The liver transplant-providing institutes in Pakistan are mainly established in the private sector. A considerable proportion of the Pakistani population is poor and are unable to afford this costly procedure. As a result, these patients are just offered supportive treatment, and unluckily they die without undergoing liver transplantation.

Also, the cost of a liver transplantation procedure in Pakistan does not include the cost of screening, physician consultation fees, pre-transplant diagnostics laboratory investigations, management of various post-transplant complications, and long-term expenses on immunosuppression use [2]. The immunosuppressant drugs are costly. The approximate cost is USD 200–300/month or even more. It is rarely possible that patients will not need immunosuppressants in the long term. Besides all this, some old patients may also need management of their comorbid illness. Also, families have to pay for the indirect expenses, like transportation and staying near the hospital during their patient procedures [3].

Many patients initially succeed to undergo liver transplantation but fail to do regular follow and ultimately die because of rejection or other post-transplant complications. So, before proceeding with liver transplantation the risk to benefit needs to be weighed for every patient. Every recipient and donor should be screened on psycho-social and financial grounds. They should be offered liver transplants only if they seem capable of doing a proper follow-up and meet the expenses for post-transplant complications. The government should build more public sector institutes for liver transplantation and should allocate more funds to the already established centres for the free treatment of these patients. It is also critical to build collaboration with other financing agencies and non-governmental groups to provide funds for such needy patients.

## Ethical approval

N/A.

## Sources of funding

None.

## Author contribution

Kaleem Ullah: Concept, Manuscript Writing, Literature Review. Abdul Wahab Dogar: Drafting, Final Approval. Husna Nisar: Literature Review. Sidhant Ochani: Review-Editing, Referencing. Afnan W.M. Jobran: Literature Review. Muhammad Wajeeh Nazar: Review-Editing.

## Registration of research studies

N/A.

## Guarantor

Sidhant Ochani.

## Consent

N/A.

## Declaration of competing interest

None.
